# Mode Choice between Private and Public Transport in Klang Valley, Malaysia

**DOI:** 10.1155/2014/394587

**Published:** 2014-02-19

**Authors:** Onn Chiu Chuen, Mohamed Rehan Karim, Sumiani Yusoff

**Affiliations:** Center for Transportation Research, Faculty of Engineering, University of Malaya, Lembah Pantai, Malaysia

## Abstract

In 2010, Klang Valley has only 17% trips each day were completed using public transport, with the rest of the 83% trips were made through private transport. The inclination towards private car usage will only get worse if the transport policy continues to be inefficient and ineffective. Under the National Key Economic Area, the priority aimed to stimulate the increase of modal share of public transport in the Klang Valley to 50% by 2020. In the 10th Malaysia Plan, the Klang Valley Mass Rapid Transit was proposed, equipped with 141 km of MRT system, and will integrate with the existing rail networks. Nevertheless, adding kilometers into the rail system will not help, if people do not make the shift from private into public transport. This research would like to assess the possible mode shift of travellers in the Klang Valley towards using public transport, based on the utility function of available transport modes. It intends to identify the criteria that will trigger their willingness to make changes in favour of public transport as targeted by the NKEA.

## 1. Transportation in Malaysia

The major mode of transport in Malaysia is by road which is predominantly influenced by Malaysia's geographical characteristics. Malaysia consists of two geographical regions divided by the South China Sea. With around 330,000 square kilometers of total land area, the road network is a valuable asset and acts as a catalyst to the economic and social growth of the country [1]. In today's world, where globalization continues to develop, the establishment of a well-maintained road network and infrastructure will boost the nation's competitiveness and maintain an advantage over its rivals.

However, it must be regretful to acknowledge that the Malaysian transportation sector is unsustainable especially with the rapid rise of car numbers and usage on the road. The numbers of vehicles in the country have increased tremendously in the past two decades. The numbers quadrupled from as little as 5 million in 1991 to 21.4 million in 2011 with an average annual growth rate of 7.5%. The growth in the number of vehicles in the country has been 3.3 times faster than the growth in the population, which had an average 2.25% annual growth rate in the same period [2–5].

Economic growth and the growing demand for mobility in developing countries are leading to an increased amount of passenger car ownership [6]. This has led to congestion and consequently increases the carbon emission. In 2010, Malaysia recorded the highest number of passenger car ownerships at 311 per 1000 people. It is a massive jump since 1990, where it was only 91 in 1990, 133 in 1996, and 210 in 2002 [5, 7–9].

## 2. Study Area

**Figure 1:**
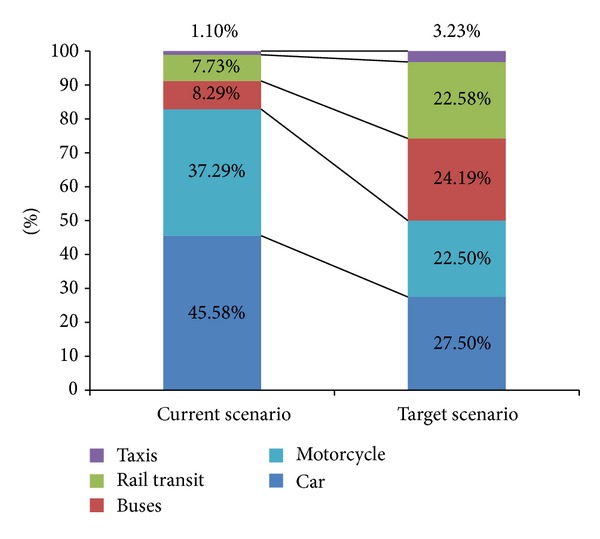
Trip ratio in current scenario versus target scenario by 2020.

Klang Valley including the Federal Territory of Kuala Lumpur, Selangor district of Petaling, Klang, Gombak, and Hulu Langat, comprises a total population of 6.187 million in 2010, and this is expected to grow to 10 million by 2020, located in the central of peninsular Malaysia (Figure 1) [10].

In 2010, Klang Valley contributed around RM263 billion to the country's gross national income (GNI), which translates into 30 percent of the nation's GNI in that year. With only 20 percent of the national population, the contribution demonstrates the importance of Klang Valley to the nation's economic growth [11].

### 2.1. Public Transportation Services in Klang Valley

Public transport in the Klang Valley covers a variety of modes such as bus service, rail transport, and a taxi service. Bus is the most important public transport service in Klang Valley. Buses have the most passengers among the public transport service and make approximately 600,000 trips each day. In 2011, almost 60% of Klang Valley's populations lived within 400 meters of a bus route, and the coverage is expected to increase up to 70% by 2012, as the priority is to deliver 50% of the share in public transportation in the Klang Valley [12, 13]. The public rail transportation in the Klang Valley is fairly extensive and has a total distance coverage of 224.6 km. The public rail services make a total of 560,000 trips daily and they have 7 main routes.

### 2.2. Congestion in Klang Valley

Just as the United States has New York, the United Kingdom has London, China has Beijing, and Japan has Tokyo, so Malaysia has Kuala Lumpur as an iconic city that has been the pillar of the national economic growth and the landmark of our country. Malaysia aims to achieve high income status, and the contribution from its primary city is significantly needed to realize this growth [11]. However, urbanization, at the same time, brings a strain to the city and its citizens. Traffic congestion, limited parking space, and environmental pollution have become prominent concern in Klang Valley, especially with the enormous growth of motorization in that region [14].

In 2010, in Klang Valley, only 17% or approximately 1.24 million trips each day were completed using public transport (Table 1). The remainder of the 83% or 6 million trips were made using private transport with single occupancy vehicles (SOVs) topping the list [15, 16].

The main reason behind the poor public transportation usage and overdependency on private cars is because most travellers prefer cars that are more cost- and time-effective than an unplanned public transport system [14].

In the 10th Malaysia Plan, the Klang Valley Mass Rapid Transit was proposed, equipped with 3 lines of Mass Rapid Transit (MRT) system measuring 141 km in the Klang Valley. The Klang Valley MRT will integrate with the existing rail networks such as light rail transit (LRT), Monorail, KTM commuter, and bus services (inter/intra) to form an effective and efficient public transportation system. The building of MRT is expected to ease the deteriorating traffic congestion in the Klang Valley by extending the currently inadequate rail network system. It is estimated that a single 4-carriage MRT train will be able to carry 1,200 passengers, and it is equal to the average number of passengers carried by 700 cars [15, 17]. Nevertheless, adding kilometers into the rail system will not help, if people do not make the shift from private into public transport.

There are currently approximately 6.187 million people in the Klang Valley and this number is expected to grow up to 10 million by 2020, which comprises almost one-third of Malaysia's existing population. The Klang Valley has 3.2 million private cars and this number is growing exceptionally fast with an average of 30,000 cars per month. The modal share of private cars will continue to rise, and it is expected to reach 7 million by 2020. The inclination towards private car ownership will only get worse if the transport policy continues to be inefficient and ineffective. Currently, the major roads surrounding the city centre are nearing their usage capacity and the issue of land scarcity in Klang Valley hinders the building of more roads and more parking facilities. Cars are being double- and triple-parked on the road, causing even more congestions. Traffic congestion in the city is increasing and it is taking longer to get to one's destination, making the situation for revamping the urban transport system even more critical. Unless there is a significant shift to more sustainable public transport, by 2020, Klang Valley will not be able to sustain 7 million private cars [15, 16, 18]. Besides, the productive time lost due to road congestion will eventually cost the nation its competitiveness especially in its key economic corridor. Moreover, the situation is especially concerning when comparing the public transport usage in Klang Valley with cities such as Singapore, Hong Kong, and London, where the proportion of public transport trips is 64%, 74%, and 90%, respectively.

Under the Economic Transformation Programme, for the next decade, the National Key Economic Area (NKEA) aimed to stimulate the increase of modal share of public transport in the Klang Valley to 50% from 18% and place Kuala Lumpur and Klang Valley among top 20 livable cities globally in terms of economic growth by 2020, as outlined in the Greater Kuala Lumpur/Klang Valley (GKL/KV) National Key Economic Area (NKEA) [11]. The GKL/KV that comprises the area under the administration of ten local authorities is one of the 12 NKEAs under the Economic Transformation Programme. The priority goal for NKEA for the next decade is to drive economic growth in parallel with the effort to upgrade the attractiveness of the city in terms of where people would like to live [11]. A well-grounded public transport policy is needed and has to be coordinated with an effective public transport planning to push public transport in moving forward.

## 3. Factor Affecting Individual Choice for Transport

Many previous transport studies investigated the relationship among the characteristics of travelers, trips, and transport facilities, with the individual's transport mode choice behavior. They found that, in general, there are three core characteristics influencing the mode choice of individuals [19–21]:

- characteristics of the travelers such as traveler's background, household structure and income, vehicle ownership, and availability of vehicle choice;
- characteristics of the trips such as the purpose of the trip, time of the trip, and trip distance;
- characteristics of the transport facility such as travel duration, costs, quality of service, and parking space availability.

Most studies focus on the possible correlation between the individual choices of transport mode and these characteristics. However, these characteristics could be interrelated with each other and directly or indirectly affect the public transport demand in practice [22].

## 4. Choice Modelling

There will be no success in developing a sustainable transport policy, if the policymakers do not recognize the traveler's need for choice and demand. However, the most challenging task in transportation forecasting process is to identify the influencing factors on a traveller's choice.

The discrete choice models have commonly selected transport modelling for characterization of each traveller's behaviour [23]. The discrete model is represented by a theoretical framework in terms of the utilitarianism. Utilitarianism is a theory which is based on the utility maximization of a choice from a set of alternatives. The higher the utility of choice, the greater the value and benefit which the consumer will get from it and the greater the possibility that this choice will be selected.

The utility (*U*_*x*_) for alternative *x* would consist of a systematic attributes *V*_*x*_ which is a function of relevancy to decision-making process and a constant *e*_*x*_ representing the uncertainty derived from individual behavior and modeller measurement errors [24].

Therefore, the utility function for transport mode *x* can be formed from the weighted sum of a set attributes of choice, for example,

(1) $$\begin{array}{c} U_{x}=b_{1}\times {V1}_{x\;}+b_{2}\times {V2}_{x\;}+b_{3}\times {V3}_{x\;}+b_{4}\times {V4}_{x\;}+e_{x}, \end{array}$$

where*U*_*x*_ is the utility function of travel by transport *x*;  *V*1_*x*_, *V*2_*x*_, *V*3_*x*_,  and *V*4_*x*_ are attributes of transport  *x*;  *b*_1_, *b*_2_, *b*_3_,  and *b*_4_ are weight of each attribute;  *e*_*x*_ is the mode constant.

Three main choices will be modelled in this study, which are car, bus, and rail. The attributes related to the car selection are the network, parking space, parking cost, reliability, ownership cost, fuel cost, toll frequency, toll cost, traveling time, and traffic. In contrast, attributes related to the rail travel are accessibility distance, network connectivity, service frequency, transit interval, traveling time, traveling cost, reliability, parking availability, and cabin environment. For bus the attributes are accessibility distance, network connectivity, service frequency, transit interval, travelling time, traffic, traveling cost, reliability, and cabin environment. Based on the attributes listed, the initial assumptions of the utility functions for the three modes are shown in the equation below:

(2) Ucar=constantcar+b1car×car_network+b2car×car_parkingspace+b3car×car_parkingcost+b4car×car_reliability+b5car×car_price+b6car×car_fuelprice+b7car×car_tollno+b8car×car_tollcost+b9car×car_time+b10car×car_traffic,

(3) Urail=constantrail+b1rail×rail_accessdistance+b2rail×rail_network+b3rail×rail_transittime+b4rail×rail_transitno+b5rail×rail_time+b6rail×rail_cost+b7rail×rail_reliability+b8rail×rail_parking+b9rail×comfortlevel,

(4) Ubus=constantbus+b1bus×bus+accessdistance+b2bus×bus_network+b3bus×bus_servicetime+b4rail×bus_transitno+b5bus×bus_time+b6bus×bus_traffic+b7bus×bus_cost+b8bus×bus_reliability+b9bus×bus_comfortlevel.

Based on the utility function of each mode, the proportion of travellers who would use car, rail, or bus will be

(5) $$\begin{array}{c} P_{\text{car}}=\frac{e^{U_{\text{car}}}}{e^{U_{\text{car}}}+e^{U_{\text{rail}}}+e^{U_{\text{bus}\;}}},\\ P_{\text{rail}}=\frac{e^{U_{\text{rail}}}}{e^{U_{\text{car}}}+e^{U_{\text{rail}}}+e^{U_{\text{bus}}}},\\ P_{\text{bus}}=\frac{e^{U_{\text{bus}}}}{e^{U_{\text{car}}}+e^{U_{\text{rail}}}+e^{U_{\text{bus}}}}. \end{array}$$

## 5. Survey Method

Surveys have become a standard tool for statistical research in the social sciences, marketing, and government agency, with the introduction of probability sampling in 1930. Online surveys have becoming an essential research tool for data collection. The 2005 European Society for Opinion and Marketing Research (ESOMAR) report shows that the online survey is now playing an important role in market research, which accounted for 20% of expenditure on data collection methods around the world [25]. Since online survey methods have made many constructive advantages, this study implemented the online survey as its primary collection tool.

### 5.1. Sampling Size

In research, the sample size is usually outlined by the requirement of having a sufficient statistical influence and the cost of data collection. A larger size of sample often leads to an increase in accuracy when conducting statistical analysis. However, in some cases, extending the increase of sample sizes leads to minimal or no increase in the accuracy at all. Usually, sample sizes are designed in a way that is based on the estimated quality of the survey result. Confidence interval (CI) is one of the key measurements of the quality of survey. The three most common confidence intervals are 90%, 95%, and 99%. In applied practice, the 95% level is the most frequently used confidence interval. A confidence interval of 95% means that it would obtain the identical results 95% of the time [26]. The confidence interval of 95% was chosen for this study. Based on Table 2, with the confidence interval of 95%, and population of 6.187 million capita (>1 million) in the Klang Valley [10], the minimum sample size required for this survey is 384.

## 6. Survey Results

A total of 396 responses were collected, as this number fulfilled the minimum 384 of responses to achieve 95% confidence level and 5% margin of error for population sizes of 100 million and below.

### 6.1. Logistic Regression Analysis

Logistic regression combines a series of independent variables together with individual coefficients to estimate the likelihood that a certain event based on the dependent variable will take place. In the analysis of the relationship between mode choice and mode attributes, we used binomial logistic regression.

Binomial logistic regression is used to evaluate the relationships between a dichotomous dependent variable and metric or nonmetric independent variables. The dependent variables of the analysis are mode usage (car, rail, and bus) and the independent variables are the influencing attributes of each mode. The dependent variables are dichotomous and can be divided into two groups. Group “YES” contains those who use the selected mode as the main mode for daily travel and is encoded as one. Group “NOT” contains those who did not use the selected mode as the main mode for daily travel but have used them before for other purposes. This is encoded as having a zero value.

As for all regression models, the assumption is that all data follow the hypothesized linear relationship and are often used in the analysis. However, it is sometimes the case that there are outliers who are far off the regression line. For this purpose, diagnostics have to be taken to detect these unusual cases. In this research, we define the standardized residuals that are larger than ±2 as outliers.

In the initial logistic regression model for car, there are 5 cases of outliners, 8 cases for rail, and 3 cases for bus.

Since we found outliers, we will have to rerun the logistic regression analysis without selecting those outliners and to compare the accuracy rates with the original models to determine which one we will interpret. The new classification accuracy was generated for all models as shown in Tables 3, 4, and 5.

The classification accuracy for the model car that excluded outliers was 97.8% and this was better than the initial classification accuracy for the model that included all cases by 2% at 95.8%. The classification accuracy for the model rail that excluded outliers was 95.3% and this was better than the initial classification accuracy for the model that included all cases by 4.4% at 90.9%. The classification accuracy for the model bus that excluded outliers was 98.9% and this was better than the initial classification accuracy for the model that included all cases by 3.6% at 95.2%.

Removing outliers from the analysis for all models significantly improved the overall model accuracy at least by 2%. All the remaining analyses for these models will be calculated based on the output that excludes outliers.

As shown in Table 6, the model for car has 5 independent variables with *P* values that are less than the level of significance of 0.05. The probability (*P* value) of the Wald statistic for the variable [CAR_NETWORK] is 0.047, [CAR_PARKINGCOST] is 0.031, variable [CAR_PRICE] is 0.041, variable [CAR_FUELPRICE] is 0.035, and variable [CAR_TIME] is 0.042. The [CAR_NETWORK] is an ordinal variable that is coded where greater numeric values are associated with an increase in the road network of car. Therefore, this relationship would indicate that, with a better road network of car, respondents were 9 times more likely to use rail transport as their main mode of transport. The [CAR_PARKINGCOST] is an ordinal variable that is coded where greater numeric values are associated with an increase in the cost of parking. Thus, this relationship would indicate that, with a higher price of parking, respondents were 96.2% less likely to use the car as their main mode of transport. The [CAR_PRICE] is an ordinal variable that is coded where greater numeric values are associated with an increase in the cost of owning a car. Therefore, the relationship would indicate that, with a higher price of car, respondents were 90.7% less likely to use a car as their main mode of transport. The [CAR_FUELPRICE] is an ordinal variable that is coded where greater numeric values are associated with increase in the cost of fuel price. Hence, this relationship would indicate that, with a higher price of fuel, respondents were 94.8% less likely to use a car as their main mode of transport. The [CAR_TIME] is an ordinal variable that is coded where greater numeric values are associated with an increase in the duration of travelling with a car. Hence, this relationship would indicate that, with a longer travelling time with a car, respondents were 95.8% less likely to use a car as their main mode of transport.

Based on Table 7, the model for rail has 3 independent variables with *P* values that are less than the level of significance of 0.05. The probability (*P* value) of the Wald statistic for the variable [RAIL_ACCESSDISTANCE] is 0.004, variable [RAIL_TRANSITTIME] is 0.15, and variable [RAIL_TIME] is 0.022. The variable [RAIL_ACCESSDISTANCE] is an ordinal variable that is coded where greater numeric values are associated with an increase in the access distance to the rail transport. Thus, this relationship would indicate that, the nearer the distance to the rail transport system is, the respondents were 5.4 times (1/0.184 = 5.43) more likely to use rail transport as their main mode of transport. The [RAIL_TRANSITTIME] is an ordinal variable that is coded where greater numeric values are associated with an increase in the duration of transit interval for rail transport. Hence, this relationship would indicate that, with a shorter duration of transit interval for rail transport, respondents were 66 times (1/0.015 = 66.7) more likely to use rail transport as their main mode of transport. The [RAIL_TIME] is an ordinal variable that is coded where greater numeric values are associated with an increase in the travelling time with the rail transport system. Therefore, this relationship would indicate that, with a shorter traveling time, respondents were 8 times (1/0.124 = 8.06) more likely to use the rail transport as their main mode of transport.

The model for bus based on Table 8 has 4 independent variables with *P* values that are less than the level of significance of 0.05. The probability (*P* value) of the Wald statistic for the variable [BUS_ACCESSDISTANCE] is 0.026, variable [BUS_NETWORK] is 0.031, variable [BUS_SERVICETIME] is 0.026, and variable [BUS_TIME] is 0.045. The [BUS_ACCESSDISTANCE] is an ordinal variable that is coded where greater numeric values are associated with an increase in the access distance to the public bus system. Thus, this relationship would indicate that, the nearer the distance to the public bus system is, the respondents were 27.8 times (1/0.036 = 27.8) more likely to use public bus as their main mode of transport. The [BUS_NETWORK] is an ordinal variable that is coded where greater numeric values are associated with an increase in the network of the public bus system. Therefore, the relationship would propose that, with a better network for the public bus system, respondents were 52 times more likely to use public bus system as their main mode of transport. The [BUS_SERVICETIME] is an ordinal variable that is coded where greater numeric values are associated with an increase in the period of transits with the public bus. Thus, this relationship would indicate that, the lesser the waiting time with the public bus system is, the respondents were 200 times (1/0.005 = 200) more likely to use public buses as their main mode of transport. The variable [BUS_TIME] is an ordinal variable that is coded where greater numeric values are associated with an increase in the duration of traveling with the public bus system. Hence, this relationship would indicate that, with a shorter duration of traveling period with public bus system, respondents were 21 times (1/0.046 = 21.74) more likely to use the public bus system as their main mode of transport.

### 6.2. Derivation of Utility Model

As per the objective of this study, the mode choice models will be derived to forecast the modal shift. Based on the utility attributes, we can conclude that the significant variables for mode car are road network, parking cost, car price, fuel price, and travel time. The significant variables for mode rail are accessibility distance to the rail transport system, the rail transport transit time, and travel time. Finally the significant variables for mode bus are accessibility distance, the bus network, the bus transit time, and total time spend using the service. These variables and their respective coefficients which indicate the interaction of the variable within the utility function are the factors that derive the utility functions for each alternative.  Table 9 shows the statistics of these variables.

### 6.3. Formulation of the Model Structure

The discrete choice model will be able to investigate the tendency of travellers to change their travel behaviour in relation to the choice of mode available for their daily trips. The discrete choice model is developed as a logit model between the private car and public transport options such as bus and rail.

The utility functions derived from the discrete choice model help to discover the comparative attractiveness of each mode. The interaction of each attribute in a utility function of a mode is shown by its coefficients. The positive values of these coefficients apply a positive impact on the utility function, while negative values apply a negative impact.

The logistic regression on each utility modal was repeated by incorporating significant variables only. Tables 10, 11, and 12 show the results of the logistic regression.

Based on the logistic regression analysis, 3 models are established and shown below.

*Model 1*. Utility function for mode car:

(6) Ucar=1.669(Network)−2.409(ParkCost)−2.133(CarPrice)−2.277(FuelPrice)−3.069(Time)+26.934.

*Model 2*. Utility function for mode rail:

(7) Urail=−2.035  (AccDist)−1.812(TransitTime)−2.642(Time)+18.429.

*Model 3*. Utility function for mode bus:

(8) Ubus=−2.282(AccDist)+2.224(Network)−3.775(TransitTime)−1.820(Time)+14.535.

As this study would like to identify the attraction of existing options, mitigation policies can be implemented to encourage a modal shift into public transport. By taking the average values of each attribute from Table 9, we can summarize the utility of these mode in Klang Valley as bellow.

*Model 1.* Utility function for mode car:

(9) Ucar=1.669(4.1971)−2.409(2.3154)−2.133(2.5054) −2.277(2.5448)−3.069(2.6201)+26.934=9.182.

*Model 2.* Utility function for mode rail:

(10) Urail=−2.035(3.1274)−1.812(2.9811) −2.642(2.8679)+18.429=−0.914.

*Model 3*. Utility function for mode bus:

(11) Ubus=−2.282(2.6919)+2.224(3.3297)−3.775(3.1568) −1.820  (3.0324)+14.535=  −1.639.

The positive utility on the mode car implies beneficence or increased preference on the mode, while the negative utility on both public transport modes implies decreased satisfaction on these modes.

### 6.4. Modal Shift Application

Since we are evaluating the sustainable transport policy that can be taken to reduce private car usage, we did not take the car's road network connectivity and traveling time into consideration for transport policy. This is because altering this variable will mean reducing road connectivity and increasing traveling time with private cars, which is improper conduct for policies, even though they are significant variables that contribute to travellers choice making. Tables 13 and 14 show the average rating of each attribute (parking cost, car price, and fuel price) by travellers who used and who did not use car as their main mode in daily travel.

Based on the ratings, we can interpret that travellers will choose to use car when parking cost is good (1.8065), car price is good (2.1751), and fuel price is good (2.1244). Thus, by incorporating rating value, the utility of travellers choosing car as their mode is

(12) Ucaryes=1.669(4.1971)−2.409(1.8065)−2.133(2.1751) −2.277(2.1244)−3.069(2.6201)+26.934=12.069.

Travellers will choose not to use car when car parking cost is poor (4.0968), car price is poor (3.6613), and fuel price is poor (4.0161). Thus, by incorporating the rating value, the utility of travellers not choosing car as their mode is

(13) Ucarnot=1.669(4.1971)−2.409(4.0968)−2.133(3.6613) −2.277(4.0161)−3.069(2.6201)+26.934=−0.926.

Tables 15 and 16 show the average rating by travellers who used rail as their main mode in daily travel and rating of those who did not used rail as their main mode in daily travel.

Based on the ratings, we can interpret that travellers will choose to use rail when the access distance to the system is good (2.0909), service interval is good (1.9818), and traveling time with rail is good (1.9091). Thus, the utility of travellers choosing rail as their mode is

(14) Urailyes=−2.035(2.0909)−1.812(1.9818) −2.642(1.9091)+  18.429=5.539.

Travellers will choose not to use rail system when the accessibility distance to the system is poor (4.2451), duration of transit is poor (4.0588), and traveling time with rail is poor (3.920). Thus the utility of travellers not choosing rail as their mode is

(15) Urailno=−2.035(4.2451)−1.812(4.0588) −2.642(3.920)+18.429=−7.921.

Tables 17 and 18 show the average rating by travellers who used bus as their main mode in daily travel.

Based on the ratings, we can interpret that travellers will choose to use public bus when access distance to the system is good (1.6531), bus service network is good (4.1429), service interval is good (2.2959), and traveling time with bus is good (2.051). Thus, the utility of travellers choosing bus as their mode is

(16) Ubusyes=−2.282(1.6531)+2.224(4.1429) −3.775(2.2959) −1.820(2.051)+14.535=7.577.

Traveller will not use public bus when the accessibility distance to the system is poor (3.8621), bus service network is poor (2.4138), duration of transit is poor (4.1264), and traveling time with rail is poor (4.1379). Thus, the utility of travellers not choosing bus as their mode is

(17) Ubusno=−2.282(3.8621)+2.224(2.4138)−3.775(4.1264) −1.820  (4.1379)+  14.535=−12.018.

As a result, the utility of each mode is summarized in Table 19; travellers will choose car, when its utility reaches 12.069, and they will not choose it when its utility is −0.926 and below. Travellers will choose rail when its utility is more than 5.539 and will not choose it when its utility is −7.921 and below. Travellers will choose bus when its utility is more than 7.577 and will not choose it when its utility is −12.018 and below.

## 7. Scenario Modelling

The urban transport planning procedure, also referred to as the sequential four-step model, has been used to predict the flow of traffic, derived from the sequence of trip generation, trip distribution, mode choice, and the trip assignments. The urban transport planning procedure is essential in assisting the prediction of different traffic scenarios and evaluating their results based on their performance. For the purposes of this study, two scenarios are formulated. The base scenario is the current scenario based on the actual trip data from 2010. The case scenario is the forecast of the new system with the change at the mode utility phase, where a new modal split is derived after implementation of sustainable transport policies based on attractiveness of mode attributes. The transport modes included in this study are car, bus, and rails.

### 7.1. Base Scenario

In 2010, the Klang Valley had approximately 7.24 million trips daily. The data from the secondary source can be broken down as follows (Table 20) [15, 16].

The private vehicle category consists of cars and motorcycles, with the ratio of cars to motorcycles being approximately 11 : 9 [27–29]. As a result, we can assume that the breakdowns of the private vehicle type for daily trips are as shown in Table 21.

Thus, we can conclude that the usage of cars is roughly 45.6% of the total daily trips within Klang Valley.

The government is pushing to increase the rate of public transport ridership to 50% by year 2020 from 17% in year 2011 [15, 30]. We extrapolated the growth based on a linear trend in the ratio of 1 : 1 between public transport and private transport. The details are as shown in Figure 1.

In 2010, Klang Valley had 3.3 million trips made by cars and by 2020 it expects to have 7 million. Meanwhile, the population is expected to increase from 6 million people to 10 million by 2020 [15, 31].

**Figure 2:**
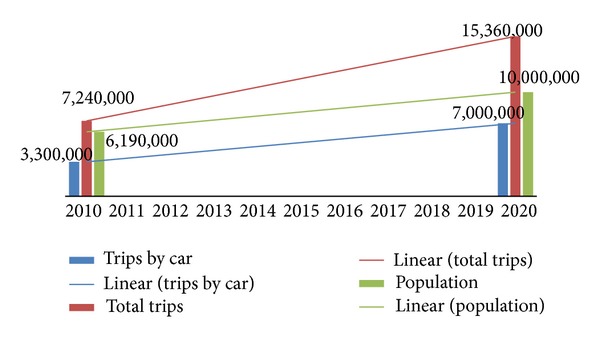
Growing trend of trips and population for Klang Valley.

**Figure 3:**
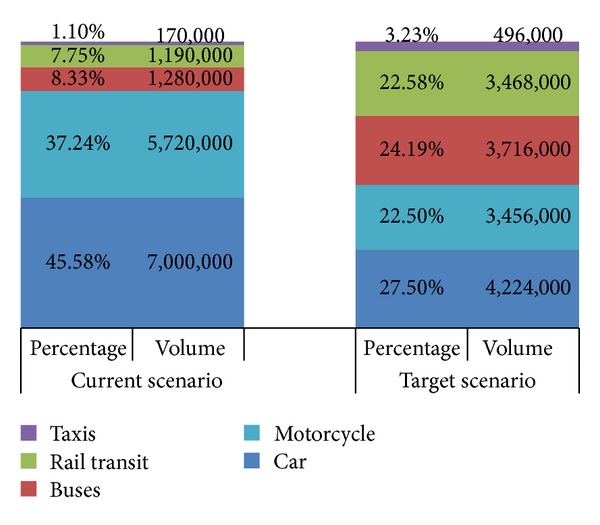
Trip volume in current scenario versus target scenario by 2020.

**Figure 4:**
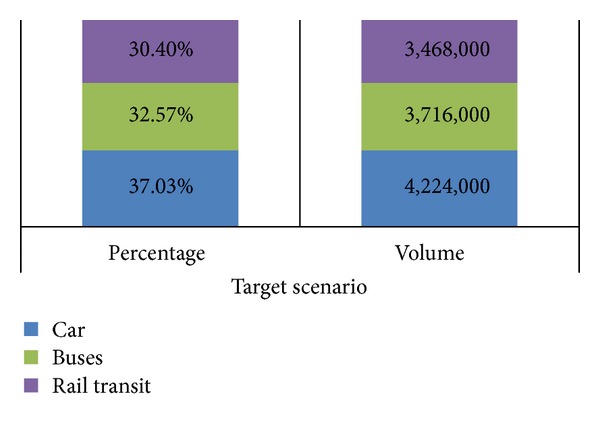
Modal split among car, bus, and rail transit for target scenario.

Based on the extrapolation at Figure 2, we expect the total daily trips to reach 15.36 million by 2020.  Figure 3 shows the target scenario where public transport mode split would increase to 50% where trips with private cars could reduce to 4.23 million from 7 million daily.  Figure 4 shows the modal split among car, bus, and rail transit for target scenario.

### 7.2. Case Scenario

Here, three scenarios were formulated based on policy measurement to identify the modal split.

**Case 1 (probability of mode shift if policy has to be taken to reduce the utility of private car)**

If we would like to push travellers into making mode change from private car into public transport, policy has to be taken to increase the parking cost, the car price, and the fuel price, so that there will be opportunity for reduction in the utility of private car from 9.182 to −0.926.

Therefore, the probability of travellers choosing selected mode among choices of car, bus, and railcan be determined by

(18) $$\begin{array}{c} P_{\text{car,bus,rail}}=\frac{e^{U_{\text{car,bus,rail}}}}{e^{U_{\text{car}}}+e^{U_{\text{bus}}}+\;e^{U_{\text{rail}}}} \end{array}$$

with *U*_car_ = − 0.926; *U*_rail_ = − 0.914; *U*_bus_ = − 1.639.

Therefore, we can conclude that the mode split of travellers is

(19) $$\begin{array}{c} P_{\text{car}}=39.96\%;\;\;P_{\text{rail}}=40.45\%;\;\;P_{\text{bus}}=19.59\%. \end{array}$$

**Figure 5:**
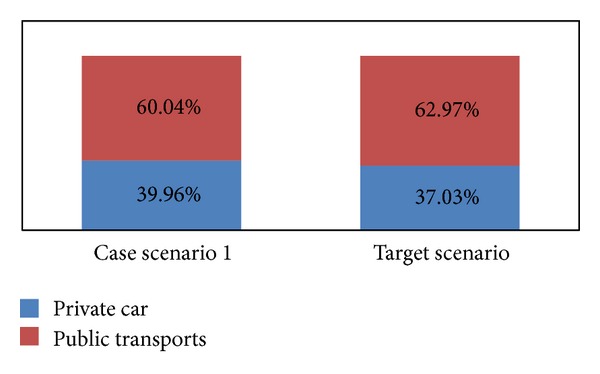
Trip ratio in case scenario 1 versus target scenario.

From scenario 1 (Figure 5), we can see that, if policy intervention is taken based on push measurement by putting extra cost on using the private transport, modal shift will happen. However, with push measurement alone, the modal shift would not reach the target volume of 37.03% for private car and 62.97% for public transport.

**Case 2 (probability of mode shift if policy has to be taken to increase utility of public transport)**

If we would like to pull travellers into making mode change from private car into public transport, policy has to be taken to reduce the access distance, the service interval, and traveling time and improve the bus network, so that there will be opportunity for increment in the utility of rail transport from −0.914 to 5.539 and bus from −1.639 to 7.577.

Therefore, the probability of travellers choosing selected mode among choices of car, bus, and railcan be determined by

(20) $$\begin{array}{c} P_{\text{car,bus,rail}}=\frac{e^{U_{\text{car,bus,rail}}}}{e^{U_{\text{car}}}+e^{U_{\text{bus}}}+e^{U_{\text{rail}}}} \end{array}$$

with *U*_car_ = 9.182; *U*_rail_ = 5.539; *U*_bus_ = 7.577.

Therefore, we can conclude that the mode split of travellers is

(21) $$\begin{array}{c} P_{\text{car}}=81.50\%;\;\;P_{\text{rail}}=2.13\%;\;\;P_{\text{bus}}=16.37\% \end{array}$$

**Figure 6:**
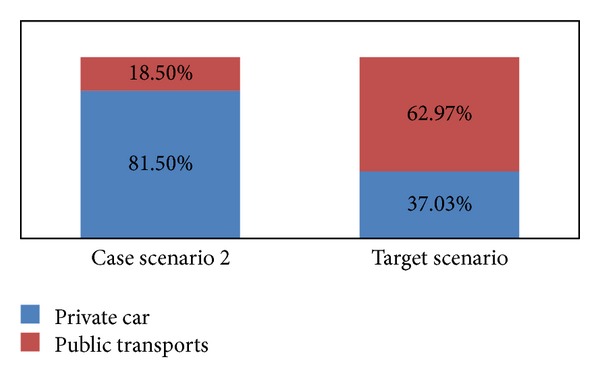
Trip ratio in case scenario 2 versus target scenario.

From scenario 2 (Figure 6), we can see that, if policy intervention is taken based on pull measurement on the public transport by reducing the access distance, the service interval, and traveling time and improving the network of bus, targeted modal shift will not happen. Car user will still choose car even if public transports are being improved.

**Case 3 (probability of mode shift if policy has to be taken to reduce utility of private car along with increase utility of public transport)**

If we reduced the utility of private car from 9.182 to −0.926, the utility of rail transport from −0.914 to 5.539 and bus from −1.639 to 7.577 will be increased.

Therefore, the modal split can be determined by

(22) $$\begin{array}{c} P_{\text{car,bus,rail}}=\frac{e^{U_{\text{car,bus,rail}}}}{e^{U_{\text{car}}}+e^{U_{\text{bus}}}+\;e^{U_{\text{rail}}}} \end{array}$$

with *U*_car_ = − 0.926; *U*_rail_ = 5.539; *U*_bus_ = 7.577.

Therefore, we can conclude that the mode split of travellers is

(23) $$\begin{array}{c} P_{\text{car}}=0.02\%;\;\;P_{\text{rail}}=11.52\%;\;\;P_{\text{bus}}=88.46\%. \end{array}$$

**Figure 7:**
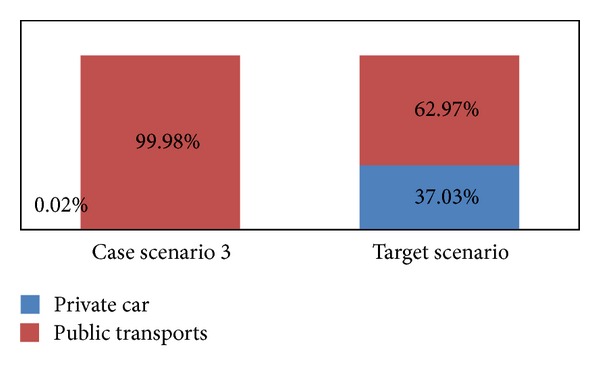
Trip ratio in case scenario 3 versus target scenario.

From scenario 3 (Figure 7), we can see that, if policy intervention is taken together on public and private transport, targeted modal shift will happen and travellers will choose public transport.

In summary, it indicates that with improvement in public transport alone it would not be enough to attract general public into making modal shift and moreover to achieve the NKEA target of 50% public transport ridership. This is an indication that, as long as the cost of private transport is still where they are now, they will still be the people choice of mode, even if improvements are made in current public transport system. Nevertheless, if policy intervention is taken on both private transport and public transport based on push and pull measurement, modal shift will occur. Thus, in order to achieve the NKEA goal, a well-grounded transport policy is needed in both public and private transports.

## 8. Summary

Economic advancement and population growth rapidly increase the trend of mobilization. Travel can be categorized by quantity of trip, trip distance, choice of mode of transport, and route selection. The mobilization of people is largely affected by the urban transport system. The urban transport system has a direct effect on the travel patterns, transport demand, and also the impact on the environment. Sustainable transport systems are those which aim to optimize the usage of fossil fuel to reduce emissions. Thus, it is vital to reduce the usage of private transport and shift the trend towards sustainable modes of transport, such as public transport.

The present research studied the current unsustainable transport system in Klang Valley. Almost 83% out of 7.23 million trips daily in Klang Valley are made using private transport and generally consist of single occupancy vehicles. If no measures are taken to reduce the ratio of private vehicles to public vehicles, with the fast growing rate of thirty thousand private cars each month, very soon Klang Valley will have to face a doubling of the number by 2020. If this happens, Klang Valley might not be able to support this capacity, signifying a state of unsustainability. Under the Greater KL National Key Economic Area, the Economic Transformation Program has set itself the target of making 50% of the mode share public transport by 2020. Research has to be taken to determine the significant factor that will encourage mode shifting, so that an effective transport policy can be taken to tackle this factor. Otherwise, travellers will keep on using their cars.

The objective of this research is to identify the potential shift of the mode of transport used by travellers towards the public transport away from private transport. A review preference survey was conducted through means of a household survey within Klang Valley. The review preference survey helped to identify the perception of the travelers towards each mode of transport. From the collected parameters of choice, discrete choice models were constructed through mode shift analysis to estimate the likelihood of travellers shifting from their current mode towards public transportation. This offers important information for policy makers to help them identify which transport attributes will be useful in making the potential shift. The use of review preference techniques with a discrete choice model offers a platform for advanced transport analysis to study people's behaviour in making choices on the basis of a hypothetical scenario. The combination of these techniques makes it possible for policymakers to gain an insight into present and future scenarios and provide policymakers with useful information for formulating policies, plans, and goals for the development of the transport sector. Three scenarios were used in this study for the modelling of urban transport incorporating the discrete choice models.

The results of this study reveal that the travellers' likelihood of choosing a car as their main mode of transport is significantly affected by parking costs, car prices, and fuel prices. While, in the case of rail transport, the accessibility distance to the rail transport system, transit time, and time spent using the service are the significant factors that will attract travellers, the accessible distance for the public bus service, as well as the bus network, buses transit time, and travelling time are the significant factors that will attract travellers.

Three scenarios have been formulated for the purpose of this study to identify the workability of the NKEA target scenario. The base scenario is based on the projection of actual trip data from 2010 and the target scenario is the forecast of the new system with a 50% public transport mode share based on the NKEA target. When analyzing the scenarios, it shows that the NKEA target could be only achieved by taking policy measurements in both public and private transports. Thus, it can be concluded that the improvement in public transport alone would not create the desired modal share as targeted by NKEA. Therefore, policy intervention in both public and private transports is needed if the NKEA targets are to be achieved.

## Figures and Tables

**Table 1:** Number of daily trips by each transport mode in Klang Valley.

Type of transport	Daily trips	Percentage
Private transport	6,000,000	83
Buses	600,000	8.3
LRT (light rail transit)	400,000	5.5
KTM commuter	100,000	1.4
Taxis	80,000	1
Monorail	40,000	0.5
ERL (express rail link)	20,000	0.3

**Table 2:** Sample sizes based on population and confidence interval.

Population	Confidence Interval		
	90%	95%	99%
	Sample size		
1,000	215	278	400
10,000	264	370	623
100,000	270	383	660
1,000,000+	271	384	664

**Table 3:** Classification table for car excluding outliners.

Observed	Predicted		
	CAR_USE		Percentage correct
	NOT	YES	
Step 1			
CAR_USE			
NOT	58	3	95.1
YES	3	215	98.6
Overall percentage			97.8

**Table 4:** Classification table for rail excluding outliners.

Observed	Predicted		
	RAIL_USE		Percentage Correct
	NOT	YES	
Step 1			
RAIL_USE			
NOT	94	5	95.1
YES	5	105	95.5
Overall Percentage			95.3

**Table 5:** Classification table for bus excluding outliners.

Observed	Predicted		
	BUS_USE		Percentage Correct
	NOT	YES	
Step 1			
BUS_USE			
NOT	86	1	98.9
YES	1	97	99.0
Overall Percentage			98.9

**Table 6:** Variables in the regression model for car.

	Coefficient	Standard error	Wald	Degree of freedom	*P* value	Exp. coefficient
Step 1						
* CAR_NETWORK *	*2.285 *	*1.149 *	*3.955 *	*1 *	*.047 *	*9.822 *
CAR_PARKINGSPACE	−.392	1.281	.094	1	.760	.676
* CAR_PARKINGCOST *	*−3.259 *	*1.507 *	*4.674 *	*1 *	*.031 *	*.038 *
CAR_RELIABILITY	−.466	1.446	.104	1	.747	.627
* CAR_PRICE *	*−2.375 *	*1.162 *	*4.174 *	*1 *	*.041 *	*.093 *
* CAR_FUELPRICE *	*−2.948 *	*1.401 *	*4.428 *	*1 *	*.035 *	*.052 *
CAR_TOLLNO	−1.272	1.006	1.598	1	.206	.280
CAR_TOLLCOST	−.420	1.323	.101	1	.751	.657
* CAR_TIME *	*−2.808 *	*1.382 *	*4.127 *	*1 *	*.042 *	*.060 *
CAR_TRAFFIC	−.041	.906	.002	1	.964	.960
Constant	37.351	18.213	4.206	1	.040	1.665*E*16

Italic font refer to variables with *P*-value less than 0.05.

**Table 7:** Variables in the regression model for rail.

	Coefficient	Standard Error	Wald	Degree of freedom	*P*-value	Exp. coefficient
Step 1						
*RAIL_ACCESSDISTANCE *	*−1.694 *	*.581 *	*8.499 *	*1 *	*.004 *	*.184 *
RAIL_NETWORK	.845	.668	1.598	1	.206	2.327
*RAIL_TRANSITTIME *	*−1.944 *	*.797 *	*5.945 *	*1 *	*.015 *	*.143 *
RAIL_TRANSITNO	−.601	.634	.899	1	.343	.548
*RAIL_TIME *	*−2.085 *	*.907 *	*5.283 *	*1 *	*.022 *	*.124 *
RAIL_COST	.887	1.045	.720	1	.396	2.427
RAIL_RELIABILITY	−.504	.689	.535	1	.464	.604
RAIL_PARKING	.723	.822	.774	1	.379	2.061
RAIL_COMFORTLEVEL	−.042	.600	.005	1	.944	.959
Constant	12.665	6.614	3.667	1	.056	316478.785

Italic font refer to variables with *P*-value less than 0.05.

**Table 8:** Variables in the regression model for bus.

	Coefficient	Standard Error	Wald	Degree of freedom	*P*-value	Exp. coefficient
Step 1						
*BUS_ACCESSDISTANCE *	*−3.335 *	*1.494 *	*4.980 *	*1 *	*.026 *	*.036 *
*BUS_NETWORK *	*3.965 *	*1.841 *	*4.637 *	*1 *	*.031 *	*52.704 *
*BUS_SERVICETIME *	*−5.343 *	*2.406 *	*4.933 *	*1 *	*.026 *	*.005 *
BUS_TRANSITNO	*−*1.914	1.478	1.676	1	.195	.148
*BUS_TIME *	*−3.085 *	*1.542 *	*4.005 *	*1 *	*.045 *	*.046 *
BUS_TRAFFIC	2.017	1.633	1.525	1	.217	7.517
BUS_COST	.352	1.171	.090	1	.764	1.422
BUS_RELIABILITY	2.271	1.729	1.725	1	.189	9.689
BUS_COMFORTLEVEL	*−*1.634	1.362	1.440	1	.230	.195
Constant	14.379	11.584	1.541	1	.214	1757316.086

Italic font refer to variables with *P*-value less than 0.05.

**Table 9:** Statistic of significant variables.

	Mean	Median	Mode	Std. deviation	Variance	Minimum	Maximum
CAR_NETWORK	4.1971	4.0000	5.00	.89795	.806	1.00	5.00
CAR_PARKINGCOST	2.3154	2.0000	1.00	1.24404	1.548	1.00	5.00
CAR_PRICE	2.5054	2.0000	2.00	.86028	.740	1.00	5.00
CAR_FUELPRICE	2.5448	2.0000	2.00	1.04776	1.098	1.00	5.00
CAR_TIME	2.6201	3.0000	2.00	.98865	.977	1.00	5.00
RAIL_ACCESSDISTANCE	3.1274	3.0000	2.00	1.40338	1.969	1.00	5.00
RAIL_TRANSITTIME	2.9811	3.0000	2.00	1.34186	1.801	1.00	5.00
RAIL_TIME	2.8679	2.0000	2.00	1.21660	1.480	1.00	5.00
BUS_ACCESSDISTANCE	2.6919	2.0000	2.00	1.40928	1.986	1.00	5.00
BUS_NETWORK	3.3297	4.0000	4.00	1.12019	1.255	1.00	5.00
BUS_SERVICETIME	3.1568	3.0000	2.00	1.14316	1.307	1.00	5.00
BUS_TIME	3.0324	3.0000	2.00	1.26793	1.608	1.00	5.00

**Table 10:** Regression model for car with significant variables.

	*B*	SE	Wald	df	Sig.	Exp(*B*)
Step 1						
CAR_NETWORK	1.669	.839	3.957	1	.047	5.307
CAR_PARKINGCOST	−2.409	.874	7.606	1	.006	.090
CAR_PRICE	−2.133	.849	6.317	1	.012	.118
CAR_FUELPRICE	−2.277	.917	6.168	1	.013	.103
CAR_TIME	−3.069	1.037	8.758	1	.003	.046
Constant	26.934	9.292	8.401	1	.004	4.982*E*11

**Table 11:** Regression model for rail with significant variables.

	*B*	SE	Wald	df	Sig.	Exp(*B*)
Step 1						
RAIL_ACCESSDISTANCE	−2.035	.522	15.190	1	.000	.131
RAIL_TRANSITTIME	−1.812	.577	9.876	1	.002	.163
RAIL_TIME	−2.642	.656	16.210	1	.000	.071
Constant	18.429	3.625	25.846	1	.000	1.008*E*8

**Table 12:** Regression model for bus with significant variables.

	*B*	SE	Wald	df	Sig.	Exp(*B*)
Step 1						
BUS_ACCESSDISTANCE	−2.282	.887	6.613	1	.010	.102
BUS_NETWORK	2.224	1.057	4.432	1	.035	9.246
BUS_SERVICETIME	−3.775	1.377	7.518	1	.006	.023
BUS_TIME	−1.820	.777	5.489	1	.019	.162
Constant	14.535	6.892	4.448	1	.035	2053317.454

**Table 13:** Rating by travellers who used car as their main mode.

	CAR_PARKINGCOST	CAR_PRICE	CAR_FUELPRICE
*N* Valid	217	217	217
Mean	*1.8065 *	*2.1751 *	*2.1244 *
Std. Deviation	.84402	.48756	.69934
Variance	.712	.238	.489
Minimum	1.00	1.00	1.00
Maximum	5.00	4.00	4.00

**Table 14:** Rating by travellers who did not use car as their main mode.

	CAR_PARKINGCOST	CAR_PRICE	CAR_FUELPRICE
*N* Valid	62	62	62
Mean	*4.0968 *	*3.6613 *	*4.0161 *
Std. Deviation	.61962	.88602	.66510
Variance	.384	.785	.442
Minimum	2.00	1.00	2.00
Maximum	5.00	5.00	5.00

**Table 15:** Rating by travellers who used rail as their main mode.

	RAIL_ACCESSDISTANCE	RAIL_TRANSITTIME	RAIL_TIME
*N* Valid	110	110	110
Mean	*2.0909 *	*1.9818 *	*1.9091 *
Std. Deviation	.79615	.72923	.47978
Variance	.634	.532	.230
Minimum	1.00	1.00	1.00
Maximum	4.00	4.00	3.00

**Table 16:** Rating by travellers who did not use rail as their main mode.

	RAIL_ACCESSDISTANCE	RAIL_TRANSITTIME	RAIL_TIME
*N* Valid	102	102	102
Mean	*4.2451 *	*4.0588 *	*3.9020 *
Std. Deviation	.99937	.96291	.87325
Variance	.999	.927	.763
Minimum	1.00	2.00	2.00
Maximum	5.00	5.00	5.00

**Table 17:** Rating by travellers who used bus as their main mode.

	BUS_ACCESSDISTANCE	BUS_NETWORK	BUS_SERVICETIME	BUS_TIME
*N* Valid	98	98	98	98
Mean	*1.6531 *	*4.1429 *	*2.2959 *	*2.0510 *
Std. Deviation	.65962	.53727	.64551	.61548
Variance	.435	.289	.417	.379
Minimum	1.00	2.00	1.00	1.00
Maximum	3.00	5.00	4.00	4.00

**Table 18:** Rating by travellers who did not use bus as their main mode.

	BUS_ACCESSDISTANCE	BUS_NETWORK	BUS_SERVICETIME	BUS_TIME
*N* Valid	87	87	87	87
Mean	*3.8621 *	*2.4138 *	*4.1264 *	*4.1379 *
Std. Deviation	1.06937	.87007	.72824	.82367
Variance	1.144	.757	.530	.678
Minimum	1.00	1.00	3.00	2.00
Maximum	5.00	4.00	5.00	5.00

**Table 19:** Summary of utility of each mode in Klang Valley.

	Utility of choosing	Utility of not choosing
Car	12.069	−0.926
Rail	5.539	−7.921
Bus	7.577	−12.018

**Table 20:** Daily trips by each transport mode in Klang Valley.

Type of transport	Daily trips	Percentage
Private vehicle	6,000,000	83%
Buses	600,000	8.3%
Rail Transit	560,000	7.7%
Taxis	80,000	1%

Total	7,240,000	

**Table 21:** Daily trips by each private transport mode in Klang Valley.

Type of private transport	Daily trips	Percentage
Car	3,300,000	55%
Motorcycle	2,700,000	45%

Total	6,000,000	

## References

[1] Zakaria S, Sufian Z (2009). *Public Works Department Malaysia Construction and Maintenance Ensuring Road Quality in Malaysia*.

[2] Institute of Public Administration (2010). *Profile of Urban Human Capital Population Census in Malaysia (1991–2009)*.

[3] Ministry of Transport Malaysia Total Motor Vehicles by Type and State, Malaysia, Until 31st December 2011. http://www.mot.gov.my/my/Statistics/Land/SUKU%20IV%202011/JADUAL%201.2.pdf.

[4] Mustapa SI, Sin TC, Peng, Yow L Energy Efficient Pathways for the Transportation Sector in Malaysia.

[5] Number of vehicles registered on the rise The Star. http://thestar.com.my/news/story.asp?file=/2011/2/26/nation/8146407&sec=nation#1342694696130104&if_height=178.

[6] Han J, Hayashi Y (2008). Assessment of private car stock and its environmental impacts in China from 2000 to 2020. *Transportation Research Part D*.

[7] Economist Intelligence Unit Hong Kong cars: Sub-sector update. Retrieved from Economist Intelligence. http://www.eiu.com/index.asp?layout=ib3Article&article_id=587536243&pubtypeid=1112462496&country_id=1560000156&rf=0.

[8] Road Transport Department Vehicle and driver statistics. http://portal.jpj.gov.my/index.php?option=com_content&view=category&layout=blog&id=23&Itemid=118&lang=en.

[9] Shariff NM Private vehicle qwnership and transportation planning in Malaysia.

[10] Department of Statistics (2010). *Population and Housing Census of Malaysia 2010 Population Distribution and Basic Demographic Characteristics*.

[11] Ministry of Federal Territories and Urban Wellbeing Greater KL/KV comprises 10 local authorities. http://app.kwpkb.gov.my/greaterklkv/overview/.

[12] Road Transport Department Statistics for Motorcar Registrations,” from Road Transport Department Malaysia. http://portal.jpj.gov.my/en/statistik-pendaftaran-motokar.

[13] Kaos J Efficient public transport system vital, The Star. http://thestar.com.my/news/story.asp?sec=nation&file=/2012/9/26/nation/12063678#13501912666424368&if_height=752.

[14] Almselati ASI, Rahmat RAOK, Jaafar O (2011). An overview of urban transport in Malaysia. *Social Sciences*.

[15] Jemali S Getting the public transport policy right, The Edge Financial Daily. http://www.theedgemalaysia.com/features/185962-getting-the-public-transport-policy-right.html.

[16] Ministry of Finance Malaysia

[17] Land Public Transport Commission Klang Valley Mass Rapid Transit. http://www.spad.gov.my/projects/klang-valley-mass-rapid-transit.

[18] PEMANDU (2012). *Government Transformation Programme the Roadmap 2*.

[19] Ortúzar JD, Willumsen LG (2001). *Modelling Transport*.

[20] Paulley N, Balcombe R, Mackett R (2006). The demand for public transport: the effects of fares, quality of service, income and car ownership. *Transport Policy*.

[21] Waerden VDP, Timmermans H, Bérénos M (2008). *Trip Characteristics and Travelers' Willingness to Change Transport Mode in Favor of Public Transport*.

[22] Balcombe R, Mackett R, Paulley N (2004). *The Demand for Public Transport: A practical Guide*.

[23] Rich J, Holmblad PM, Hansen CO (2009). A weighted logit freight mode-choice model. *Transportation Research Part E*.

[24] McFadden D, Manski CF, McFadden DL (1981). Econometric models of probabilistic choice. *Structural Analysis of Discrete Data and Econometric Applications*.

[25] European Society for Opinion and Marketing Research The global market research 2005.

[26] Bluman AG (2011). *Elementary Statistics: A Step by Step Approach*.

[27] Ibrahim Sheikh AK, Radin Umar RS, Habshah M, Kassim H, Stevenson M, Hariza A (2006). Mode choice model for vulnerable motorcyclists in Malaysia. *Traffic Injury Prevention*.

[28] Jala I An efficient MRT system is vital to make KL a great city, The Star. http://thestar.com.my/columnists/story.asp?file=/2012/7/16/columnists/transformationblues/11659363&sec=Transformation.

[29] Yamamoto T, Morikawa T, Dissanayake D (2001). *Travel Behavior Analysis and Its Implication to Urban Transport Planning for Asian Cities: Case Studies of Bangkok, Kuala Lumpur, Manila, and Nagoya ICRA Project Report*.

[30] Board of engineers Malaysia (2012). Driving a green change. *The Ingenieur*.

[31] One vehicle for every 1.2 Malaysians. http://thestar.com.my/news/story.asp?file=/2013/2/8/nation/12686649&sec=nation#1360308665314146&if_height=443.

